# From NMDA Receptor Antagonism to Cerebrovascular Effects: The Dual Facets of Nitrous Oxide in Bipolar Depression

**DOI:** 10.1192/j.eurpsy.2025.1211

**Published:** 2025-08-26

**Authors:** C. Pinheiro Ramos, A. F. Reis, C. Batista, M. J. Freire, S. Mendes

**Affiliations:** 1 Setúbal Hospital Center, Setúbal, Portugal

## Abstract

**Introduction:**

Nitrous oxide (N2O) was first discovered in 1772 by Joseph Priestley. Since its anesthetic properties were realized in 1844, it has been used in the medical and dental fields. Nitrous oxide is also known as “laughing gas” or “nos”.

Recent literature suggests that studying N2O is important, not only for understanding the pathophysiology of bipolar depression but also for exploring its potential as a treatment.

N2O is often used recreationally for its euphoric, anxiolytic, and dissociative effects.

**Objectives:**

To better understand the role of nitrous oxide, as well as its clinical implications.

**Methods:**

A search was conducted in various databases, including PubMed.

**Results:**

N2O has two potential ways to help with bipolar depression. Firstly, it can block NMDA receptors which can be beneficial for mood stabilization. Secondly, it can widen blood vessels in the frontal brain regions which can improve blood flow and oxygenation.

Clinical studies have suggested the effectiveness of NMDA receptor antagonists in the treatment of depression. Among these studies, the use of ketamine has been a significant breakthrough in the treatment of depression.

Despite its medical uses, N2O has also gained popularity on a recreational level due to its relaxing, euphoric effects and its ability to provoke hallucinations. Unfortunately, recreational use of N2O can lead to various neuropsychiatric symptoms, such as emotional symptoms (mania, depression, anxiety), personality changes, aggressive behaviour, and psychotic symptoms.

**Conclusions:**

Further research is needed to determine if N2O is beneficial in treating bipolar depression or if its use could lead to brain dysfunction (if taken recreationally).

**Disclosure of Interest:**

None Declared

